# MRI signal intensity of anterior cruciate ligament graft after transtibial versus anteromedial portal technique (TRANSIG): design of a randomized controlled clinical trial

**DOI:** 10.1186/s12891-016-1183-8

**Published:** 2016-08-10

**Authors:** Simeon J. S. Ruiter, Reinoud W. Brouwer, Tim W. G. M. Meys, Cornelis H. Slump, Jos J. A. M. van Raay

**Affiliations:** 1 Department of Orthopaedic Surgery, Martini Hospital Groningen, Groningen, The Netherlands; 2 Department of Technical Medicine, University of Twente, Enschede, The Netherlands; 3 Department of Radiology, Martini Hospital Groningen, Groningen, The Netherlands; 4 MIRA Institute for Biomedical Technology and Technical Medicine, University of Twente, Enschede, The Netherlands

**Keywords:** ACL reconstruction, MRI, Transtibial, Anatomic, Graft signal intensity

## Abstract

**Background:**

There are two primary surgical techniques to reconstruct the anterior cruciate ligament (ACL), transtibial (TT) technique and anteromedial portal (AMP) technique. Currently, there is no consensus which surgical technique elicits the best clinical and functional outcomes. MRI-derived measures of the signal intensity (SI) of the ACL graft have been described as an independent predictor of graft properties. The purpose of this study is to compare the MRI derived SI measurements of the ACL graft one year after ACL reconstruction, in order to compare the outcomes of both the AMP and TT ACL reconstruction technique.

**Methods/design:**

Thirty-six patients will be included in a randomized controlled trial. Patients who are admitted for primary unilateral ACL reconstruction will be included in the study. Exclusion criteria are a history of previous surgery on the ipsilateral knee, re-rupture of the ipsilateral ACL graft, associated ligamentous injuries or meniscal tear of the ipsilateral knee, unhealthy contralateral knee, contra-indications for MRI and a preference for one of the two surgical techniques and/or orthopaedic surgeon. Primary outcome is MRI Signal intensity ratio (SIR) of the ACL graft. Secondary outcome measures are the International Knee Documentation Committee (IKDC) Knee Examination Form,the Knee injury and Osteoarthritis Outcome Scores (KOOS) and the Anterior Cruciate Ligament OsteoArthritis Score (ACLOAS). Differences between MRI SIR assessment with the current MRI protocol (proton density weighted imaging protocol) and the additional T2*-weighted gradient-echo protocol will be assessed.

**Discussion:**

There is no consensus regarding the TT or AMP ACL reconstruction technique. SI measurements with MRI have been used in other clinical studies for evaluation of the ACL graft and maturation after ACL reconstruction compared to clinical and functional outcomes. This randomized controlled trial has been designed to compare the TT technique with the AMP technique with the use of MRI SI of the graft after ACL reconstruction.

**Trial registration:**

Netherlands Trial Registry NTR5410 (registered on August 24, 2015).

## Background

Rupture of the anterior cruciate ligament (ACL) is a frequently seen (sport) injury mostly induced by a non-contact deceleration motion. The risk of primary ACL rupture has been reported to occur in 1.5 to 1.7 % in healthy athletic population [1]. The ACL provides anterior-posterior and rotational stability to the knee joint. Initially, a rupture of the ACL leads to a synovial layer in the knee joint. The ends of the ruptured ACL tears will shrink and as a result of that, there is no healing process of the ACL. Long term consequences are chronic knee instability and osteoarthritis [2, 3].

There are several surgical techniques to reconstruct the ACL, which vary in graft type, number of bundles, method of fixation and tunnel position. More than 5000 ACL reconstructions are performed in The Netherlands each year [4]. The focus of ACL reconstruction is now changing toward the tunnel position of the ACL grafts since there is no established “gold-standard” technique for ACL reconstruction [5, 6]. The most frequently performed surgical technique is the transtibial (TT) drilling technique in which a femoral tunnel is drilled through the tibial tunnel. In this way, the femoral tunnel is placed non-anatomically relative to the native femoral ACL footprint [7]. Another surgical technique is the anteromedial portal (AMP) anatomic drilling technique which principle is based on restoring the native insertion site anatomy on both tibial and femoral side. The choice of drilling technique is depending on several factors such as surgeon experience, available equipment, patient age, skeletal maturity, body habitus, activity level and graft choice [5].

Robin et al. conducted a systematic review of the advantages and disadvantages of both reconstruction techniques based on 27 articles [5]. The main advantages of TT technique are single incision, isometric graft throughout knee range of motion and minimal tunnel and intercondylar notch impingement. However, disadvantages are that the tibial tunnel dictates the femoral tunnel, vertical and anterior graft placement, increased biomechanical demand during rehabilitation and TT requires notchplasty for visualization.

AMP technique leads to anatomic placement of femoral tunnel and independently tibial tunnel, anteroposterior stability, faster return to activity and useful for revision ACL reconstruction [5]. The main disadvantages of AMP are difficulty visualization in hyperflexion, posterior-wall blowout, technically demanding, limit fixation techniques due to short sockets, increased risk of injury to peroneal nerve and extension loss during stance phase [5].

Currently, there is no consensus which surgical technique elicits the best clinical outcomes. However, TT is the technique until now with long term follow-up results [5, 8].

Success of the ACL reconstruction can be measured by clinical, functional and patient-oriented outcome assessments include physical examination and patient-oriented questionnaires. However, these assessments are an indirect measure of the graft integrity and require large numbers of patients to detect differences between both operation techniques [9]. There is a need for a quantitative in vivo measurements method for the evaluation of the biomechanical performance and integrity of the ACL graft.

Magnetic resonance imaging (MRI) is the most effective and accurate technique for the quantitative in vivo assessment of the ACL with a sensitivity and specificity of more than 90 % [10]. Most common used MRI sequences for assessment of the ACL are turbo spin echo (TSE) sequences such as proton density weighted imaging (PDWI). Normally, an intact ACL appears in all planes as a low-signal linear band with an orientation at least as steep as the intercondylar roof. Between the fibers of the ACL there can be fat or fluid and therefore the ACL is not completely black on PDWI images. Primary signs of ACL rupture are increased signal on MRI, fiber discontinuity, abnormal orientation and undetectable fibers [11].

MRI-derived measures of the signal intensity (SI) of the ACL graft have been described as an independent predictor of graft properties [9]. Lower SI indicates low water and fat content and thus better maturity of the graft. MRI assessment with PDWI fails to correlate SI with actual graft function. A more promising technique is T2*-weighted gradient-echo MRI imaging which has been reported as a useful imaging modality to assess graft integrity [12]. The signal intensity ratio (SIR) can be calculated by:

$$ \mathrm{SIR} = \mathrm{A}\mathrm{C}\mathrm{L}\ \mathrm{graft}\ \mathrm{intensity}\ /\ \mathrm{posterior}\ \mathrm{cruciate}\ \mathrm{ligament}\ \left(\mathrm{P}\mathrm{C}\mathrm{L}\right)\ \mathrm{intensity}. $$

The purpose of this study is to compare the MRI derived SI measurements of the ACL graft one year after ACL reconstruction, in order to compare the graft integrity of both the AMP and TT ACL reconstruction technique. The anatomical AMP reconstruction technique restores rotational stability. Non-anatomical TT reconstruction technique restores anterior/posterior stability but lesser rotational stability [5]. It is hypothesized that the AMP technique will result in significantly lower MRI signal intensity one year postoperatively and thereby better graft maturity due to the better rotational stability compared to the TT technique. The results of this study may clarify which surgical technique will reveal the best clinical, functional and quantitative MRI outcomes. This paper reports on the study design of the TRANSIG (TRanstibial vs ANteromedial portal technique SIGnal Intensity) trial.

## Methods/design

### Study design

The study design is a randomized controlled trial: patients will be randomly allocated to undergo ACL reconstruction by means of the TT and AMP drilling technique. The study will be conducted at the department of Orthopaedics of the Martini Hospital Groningen, the Netherlands. The orthopaedic surgeons who are participating in this study (N = 2) are experienced in performing the TT and AMP reconstruction technique. Patients in the TT group will be operated by J.J.A.M. van Raaij. Patients in the AMP group will be operated by R.W. Brouwer. They will perform all ACL reconstructions in the present study. Two independent radiologists will evaluate MRI outcomes. One independent investigator, not involved with enrolment or surgical procedure, will evaluate the functional and clinical outcome measurements. The study has been approved by a Medical Ethics Committee, and is also registered in the Netherlands Trial Registry [Reference: NTR5410].

### Study sample

Patients suitable for enrolment in this study are candidates for primary unilateral ACL reconstruction with an age between 18 and 50 years and a proven ACL rupture confirmed by means of arthroscopy or MRI scan. Exclusion criteria are a history of previous surgery on the ipsilateral knee, re-rupture of the ipsilateral ACL graft, associated ligamentous injuries or meniscal tear of the ipsilateral knee, unhealthy or symptomatic contralateral knee by means of previous injury and/or surgery, contra-indications for MRI and a preference for one of the two surgical techniques and/or orthopaedic surgeon.

### Intervention

The anaesthetic and analgesic protocol will be standardized. A first physical examination of the knee under anaesthesia and a diagnostic arthroscopy via an anterolateral portal are performed prior to any reconstruction. In both study groups, the same graft type will be used. The hamstring tendon graft will be harvested through a 6–7 cm long anterior central skin incision. A notchplasty will always be performed to avoid graft impingement.

#### Transtibial technique

An anterolateral and anteromedial portal are used. After removing the ACL remnants and performing a notchplasty, the tibial tunnel is prepared first. The remaining stump of the ACL is the major orientation guide. The tip of the tibial aimer is placed in the posterior fibers of the ACL footprint. With a 2.4 mm guide wire, the tibial plateau is protruded. The angle of the aimer is dependent on the length of the graft. To achieve a longer tibial tunnel, the angle of the tibial aimer is increased. The tibial tunnel is drilled with a cannulated drill bit that matches the graft diameter.

The femoral tunnel is prepared through the tibial tunnel with the knee flexed at 90 degrees. With the femoral aimer, the over the top position is determined with the offset hook. With a 2.7 mm guide wire, the lateral femoral cortex is penetrated. With an endoscopic cannulated drill bit, the femoral socket is created that matches the graft diameter. The depth of the femoral socket is regulated according to the desired insertion length. The depth is 9–10 mm greater than the desired graft insertion to allow the endobutton (Smith & Nephew, Memphis, Tenn) device to rotate. With a 4.5 mm endoscopic drill bit, a transfemoral channel is created allowing the endobutton device to pass. With an endobutton depth gauge the total length of the femoral condyle is measured. After graft preparation the endobutton CL device length is determined by the difference between the total femoral channel length hand the desired femoral graft insertion length. The graft is pulled through the tibial and femoral tunnel. The endobutton device is passing through the lateral femoral cortex. Afterwards, the graft is pulled back locking the endobutton on the lateral femoral cortex. After pretensioning the graft, the tibial part is fixed with a peek interference screw (Smith & Nephew, Memphis, Tenn) with the knee in 20 degrees of flexion.

#### Anteromedial portal technique

The femoral tunnel is created first using an accessory anteromedial portal. The insertion and remnants of the ACL on the femoral side are determined. When the soft tissue ACL remnants cannot be identified, the insertion is determined just below and inferior to the lateral intercondylar ridge. The femoral tunnel is prepared in the same way as in the TT technique working through the accessory anteromedial portal. During preparation of the femoral tunnel, the knee remains flexed to 120–130 degrees. After preparing the femoral tunnel, the tibial tunnel is created compared to the TT. Femoral side is fixed with the endobutton device, tibial side with a peek interference screw with the knee in 20 degrees of flexion.

### Main study parameter/endpoint

The primary objective of this study is to conduct a randomised controlled trial to determine differences in outcomes of both the AMP and TT ACL reconstruction technique by means of the MRI SIR of the ACL graft.

### Secondary study parameters/endpoints

Differences in functional and clinical outcomes between the AMP and TT ACL reconstruction technique will be assessed by means of the standardized physical examination and recorded by the use of the International Knee Documentation Committee (IKDC) Knee Examination Form [13]. The examination consist of Effusion, Passive Motion Deficit, Ligament Examination (Lachman and Pivot Shift), Compartmental Findings, Harvest Site Pathology and Functional Test (one leg hop test). Laxity measurements will be performed by using KT-2000 arthrometer testing (MedMetric, San Diego, CA). Power measurements of quadriceps and hamstring muscle groups will be performed by using Cybex isokinetic dynamometer (HUMAC). Patient’s will be classified in four groups depending on the lowest grade of the examination form.

Differences in patient-oriented outcomes between the AMP and TT ACL reconstruction technique will be assessed by the use of the Knee injury and Osteoarthritis Outcome Score (KOOS). The KOOS is a knee-specific instrument to assess the patients’ opinion in five subscales of Pain, other Symptoms, Function in daily living (ADL), Function in Sport and Recreation (Sport/Rec) and knee-related Quality of Life (QOL) with scores ranging from 0 (worst) to 100 (best) [14].

Besides differences between MRI SIR assessment with the current MRI protocol (PDWI and PDWI SPAIR imaging protocol) and the additional T2*WI gradient echo protocol. Integrity of cartilage, menisci, bone and other ligamentous structures will be assessed by using the Anterior Cruciate Ligament OsteoArthritis Score (ACLOAS) [15, 16].

### Randomization

**Fig. 1:**
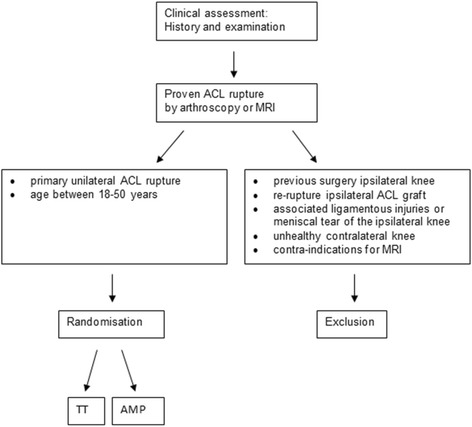
Flowchart of patient inclusion

Patients who meet the inclusion criteria will be informed about the study by their orthopaedic surgeon at the department of Orthopaedics of Martini Hospital Groningen (Fig. 1). Patients will be informed about the treatment, the risk of complications according to the Dutch Medical Treatment Contracts Act.

A random allocation set of the type of ACL reconstruction technique will be generated by means of a computer. These allocations are then sealed in consecutively numbered opaque envelopes. Once the patient has given consent to be included in the trial, the ACL reconstruction technique is then randomly assigned by opening the next sealed envelope.

Patients will be blinded for the allocated surgical technique. Measurements and data-analysis will be performed by an independent investigator.

### Data collection methods

MRI images will be obtained one year postoperatively. The current protocol for ACL assessment with MRI will be conducted with the following sequences: sagittal PDWI; coronal PDWI and sagittal PDWI SPAIR fat-suppression (SPectral Attenuated Inversion Recovery) sequence. Besides the normal knee MRI protocol, also gradient echo sequence T2*WI will be obtained by MRI by the following imaging protocol: Pulse sequence, gradient echo; repetition time (TR)/echo time (TE), 500/18 ms; slice thickness/intersection gap 4.0/1.0 mm; field of view (FOV), 15 cm (Table 1). All MRI assessments of the knee are performed on 1.5 T Philips Ingenia MRI (Philips, Best, the Netherlands) with the use a 16 elements knee coil. MRI images of the ACL graft will be analysed a radiologist. It is not possible to blind the radiologist for the allocated surgical technique since it is possible to distinguish these techniques on MRI images. Regions of interests (ROI) will be set manually on the ACL grafts with the PCL serving as control. Mean SI of the ROI for both the ACL graft and PCL will be measured. Hereafter, SIR will be calculated by:

**Table 1:** Imaging parameters

	Current knee MRI protocol		Additional MRI protocol
	PDWI	PDWI SPAIR	T2*WI
TR (ms)	1800	3310	500
TE (ms)	200	20	18
Slice thickness (mm)	3.0	3.0	4.0
Intersection gap (mm)	0.3	0.3	1.0
FOV (cm)	15	15	15

*PDWI* proton density weighted imaging, *SPAIR* SPectral Attenuated Inversion Recovery, T2*WI T2*-weighted imaging, *TR* repetition time, *TE* echo time, *FOV* field of view

$$ \mathrm{SIR} = \mathrm{A}\mathrm{C}\mathrm{L}\ \mathrm{graft}\ \mathrm{S}\mathrm{I}\ /\ \mathrm{P}\mathrm{C}\mathrm{L}\ \mathrm{S}\mathrm{I}. $$

Each SIR measurement will be repeated three times for intraobserver variation analysis and calculation of mean values. Patients will have regular follow-up with outpatient clinic appointments. Additional outpatient clinic appointment preoperatively and one year postoperatively will be conducted to assess the clinical, functional and patient-oriented outcomes of the ACL reconstruction be means of standardized physical examination, recorded by IKDC examination form and KOOS assessment. These assessments will be obtained by an independent investigator.

### Sample size calculation

Hakozaki et al. assessed the SIR of the double-bundle ACL graft 12 months after ACL reconstruction with the AMP technique [12]. On MRI, the SIR of the graft 12 months postoperative was measured of 61 patients. The mean SIR of the ACL graft was significantly higher (*p* = 0.014) in patients with positive pivot-shift test (SIR = 1.46) than in patients with negative pivot shift test (SIR = 1.25). In the present study, a difference of 20 % was considered clinically relevant. Hence, to detect a difference of 0.25 in SI (SD 0.21) with a power of 90 % and an alpha of 5 %, a sample size of 15 patients in each study group is required. To compensate for potential loss to follow-up of 20 %, the total sample size is set at 36 patients.

### Statistical analysis

All statistical analysis will be computed using SPSS (IBM SPSS, Inc., Version 22.0, 2014). Student’s *t*-test (in case of normal distribution) or Mann–Whitney *U* test (in case of non-normal distribution) will be used to assess the differences in SIR between the study and control group. To assess the differences in outcomes of the IKDC examination form between the study and control group, Chi-square test will be used. Student’s *t*-test (in case of normal distribution) or Mann–Whitney *U* test (in case of non-normal distribution) will be used to compare the outcomes of the KOOS assessments between the study and control group.

Linear regression analyses will be performed to assess whether there is a relation between SI with both MRI protocols (PDWI/PDWI SPAIR and T2*WI) and IKDC score. Linear regression analyses will also be performed to assess whether there is a relation between SIR with both MRI protocols (PDWI/PDWI SPAIR and T2*WI) and KOOS score.

## Discussion

SI measurements with MRI has been used in other clinical studies for evaluation of the ACL graft and maturation after ACL reconstruction compared to clinical and functional outcomes [9, 12, 17, 18]. However, none of these studies used SI to compare the TT technique with the AMP technique. Also, some of these studies examined the feasibility of T2*WI. The T2*WI sequence was more strongly associated with clinical outcomes (KT-2000 arthrometer) [12]. Biercevicz et al. showed also that volume and grayscale values from T2*WI MRI scans are predictive of the healing ligament [9, 18].

The anticipated results are primarily focused on graft maturity assessed by the signal intensity on the MRI SIR measurement one year after operation. It is hypothesized that in performing an anatomical ACL reconstruction, graft maturity will be better compared with the transtibial technique at one year follow up. The background of this hypothesis is the statement that the anatomical oriented ACL graft restores rotational stability in contrast to the transtibial ACL graft that only restores anteroposterior stability. Restoring the rotational stability is the keystone to a successful maturation of the ACL graft. Whether the anatomical technique will be superior to the transtibial one considering the functional results after one year follow up has to be addressed. We will use the IKDC and KOOS evaluation scores as well as the single leg hop test and AP laxity measurement. It is questionable if these parameters will be correlated with the rotational stability. A long term follow up will be necessary. With the IKDC and the KOOS evaluation and the functional tests after 2, 5 and 10 years functional outcome will be evaluated. This will be conducted in another follow up study. At longer time follow up, the risk on revision or a return of the pivot shift and other signs of instability in case of a failure will be clear in both techniques. It is important to realize that many other factors than using an anatomical or transtibial technique (timing operation, meniscal injury, level and type of sports, time when return to sports, age and sex difference man/woman) contribute to a graft failure.

To our knowledge, this is the first study which compares the TT and AMP technique for ACL reconstruction by the use of MRI SI.

## Conclusion

This paper describes the design of a randomized controlled trial on the SI of the graft after TT and AMP ACL reconstruction. The results of this study may clarify which surgical technique will reveal the best clinical, functional and quantitative MRI outcomes.

## Abbreviations

ACL, anterior cruciate ligament; AMP, anteromedial portal; IKDC, International Knee Documentation Committee; KOOS, knee injury and osteoarthritis outcome score; MRI, magnetic resonance imaging; PCL, posterior cruciate ligament; PDWI, proton density weighted imaging; ROI, region of interest; SI, signal intensity; SIR, signal intensity ratio; T2*WI, T2*-weighted imaging; TT, transtibial
